# Antiviral Intervention of COVID-19: Linkage of Disease Severity with Genetic Markers FGB (rs1800790), NOS3 (rs2070744) and TMPRSS2 (rs12329760)

**DOI:** 10.3390/v17060792

**Published:** 2025-05-30

**Authors:** Maksym Sokolenko, Larysa Sydorchuk, Alina Sokolenko, Ruslan Sydorchuk, Iryna Kamyshna, Andriy Sydorchuk, Ludmila Sokolenko, Oleksandr Sokolenko, Valentyn Oksenych, Oleksandr Kamyshnyi

**Affiliations:** 1 Department of Infectious Diseases and Epidemiology, Bukovinian State Medical University, 58012 Chernivtsi, Ukraine; sokolenko_maks@ukr.net; 2 Department of Family Medicine, Bukovinian State Medical University, 58012 Chernivtsi, Ukraine; lsydorchuk@ukr.net (L.S.);; 3 Department of Surgery No 2, Bukovinian State Medical University, 58012 Chernivtsi, Ukraine; 4 Department of Medical Rehabilitation, I. Horbachevsky Ternopil National Medical University, 46001 Ternopil, Ukraine; 5 Donauklinik, 89231 Neu Ulm, Germany; 6 Department of Medical and Biological Fundamentals of Physical Culture, Pavlo Tychyna Uman State Pedagogical University, 20300 Uman, Ukraine; 7 Department of Infectious Diseases, Bukovinian State Medical University, 58012 Chernivtsi, Ukraine; 8 Broegelmann Research Laboratory, Department of Clinical Science, University of Bergen, 5020 Bergen, Norway; 9 Department of Microbiology, Virology, and Immunology, I. Horbachevsky Ternopil National Medical University, 46001 Ternopil, Ukraine

**Keywords:** COVID-19, antiviral treatment, genes, polymorphism, FGB (rs1800790), NOS3 (rs2070744), TMPRSS2 (rs12329760)

## Abstract

The purpose of this study was to investigate polymorphic variants of the genes FGB (rs1800790), NOS3 (rs2070744) and TMPRSS2 (rs12329760) in patients with SARS-CoV-2 and to determine their role in the COVID-19 severity course against the background of antiviral therapy. Real-time polymerase chain reaction (RT-PCR) was used to genotype the polymorphism of the selected genes. GS-5734 (remdesivir) was prescribed as the basic antiviral drug. Binary logistic regression confirmed a low probability of COVID-19 developing in carriers of the A-allele of the FGB gene. The highest probability of moderate and severe COVID-19 clinical forms developing was found in G-allele carriers (especially the GG genotype) of the FGB gene (rs1800790) and the T-allele of the TMPRSS2 gene (rs12329760). Antiviral drug GS-5734 (remdesivir) administration with anti-inflammatory therapy reduces the TMPRSS2 blood level in moderate COVID-19, IL-6 in severe COVID-19 course, and fibrinogen A- and D-dimers in both groups. The proposed treatment does not significantly affect the concentration of endothelin-1, but a decrease in procalcitonin associated with additional antibacterial use was observed, especially in severe COVID-19.

## 1. Introduction

Recent studies of coronavirus-associated phenotypes (SARS, MERS and COVID-19) have shown that susceptibility to coronavirus infection and the severity of its course can also be influenced by the characteristics of the host genome [1]. The first genome-wide association study (GWAS) of COVID-19 (Severe COVID-19 GWAS Group Study) identified two loci associated with disease severity in Italians and Spaniards: the 3p21.31 loci, which contains several immune genes, and the ABO locus, which determines ABO blood groups [2]. The COVID-19 Host Genetics Initiative (HGI) was established to unite global efforts to elucidate the role of host genetic factors in susceptibility to SARS-CoV-2 and the severity of the pandemic [3]. However, reported COVID-19 genetic studies are mainly based on European populations. Therefore, it is unknown whether these findings can be applied to other populations.

Since one of the signs of severe COVID-19 is systemic coagulopathy and prothrombotic status, it was considered plausible to investigate the allelic state of the fibrinogen beta (FGB) gene (rs1800790), which, according to a number of studies, is associated with high levels of fibrinogen and D-dimers, as well as a worse prognosis for COVID-19 and high mortality [4,5].

Moreover, a recent study indicated that nitric oxide (NO) levels are markedly reduced in individuals with COVID-19, a finding that has been closely linked to the development of vascular dysfunction and immune-mediated inflammation in these patients via reactive oxygen species (ROS) elevation and decreasing NO bioavailability [6]. There are several possible pathways of endothelial NO bioavailability changes in SARS-CoV-2 infected cells: direct apoptosis of endothelial cells; SARS-CoV-2 infection associated with angiotensin-converting enzyme 2 (ACE2) decrease and leading to angiotensin II elevation, which is accompanied by the development of NO/ROS imbalance; intracellular and systemic inflammation which can cause NO/ROS imbalance in cells and tissue itself, additionally associated with mitochondrial dysfunction and accompanied by mitochondrial NO/ROS imbalance; ROS elevation changing the vascular tone and decreasing NO bioavailability [7]. All the mechanisms mentioned above provoke endothelial dysfunction in numerous organs in COVID-19 patients. Since the production and activity of endothelial NO is encoded by the corresponding gene, we decided to study the polymorphism of the endothelial NO synthase (NOS) gene in patients with COVID-19 and its association with the severity of the disease.

Notably, upon entry into the body, the SARS-CoV-2 virus interacts with cellular proteins of respiratory epithelial cells via the spike glycoprotein (S), which is first cleaved by a set of cellular proteases, including the TMPRSS2 enzyme, and is converted into the S1 and S2 subunits. The S1 part binds to the ACE2 receptor on the cell surface. The S2 part attaches to the cell membrane and can penetrate the cell. The TMPRSS2 enzyme also promotes the penetration of the virus into the cell by cleaving part of the ACE2 receptor protein [8]. TMPRSS2 activity, in turn, is encoded by the corresponding gene, which is one of the critical candidate genes responsible for the entry of SARS-CoV-2 infection into the body [9,10].

In this regard, it became necessary to investigate polymorphic variants of the genes fibrinogen beta (FGB, rs1800790), endothelial nitric oxide synthase (NOS3, rs2070744), and transmembrane serine protease 2 (TMPRSS2, rs12329760) in the context of the severity and clinical and laboratory features of COVID-19 in order to identify high-risk groups with a more severe course of the disease and a greater susceptibility to COVID-19, as well as to assess the impact of antiviral therapy.

## 2. Materials and Methods

### 2.1. Clinical–Demographic Characteristic of Patients

The cohort study involved 257 patients with COVID-19 (197—with moderate–severe COVID-19 course and 60 persons with mild course). Patients with moderate–severe COVID-19 (*n* = 197) were hospitalized at the Uman Central City Hospital, Infectious Diseases Department during 2021–2023. Diagnosis, laboratory examination, and treatment were carried out in accordance with the current protocol “Provision of Medical Care for the Treatment of Coronavirus Disease (COVID-19)” (Order of the Ministry of Health of Ukraine of 17. 05.2023 No. 913) [11], the Standards of Medical Care “Coronavirus Disease (COVID-19)” (Order of the Ministry of Health of Ukraine No. 2122 of 17.09.2020) [12], and the WHO, CDC, and global standards for the diagnosis, treatment and prevention of COVID-19 [13]. All moderate–severe patients were hospitalized in the infectious diseases department with COVID-associated community-acquired pneumonia: 21.97% (*n* = 55)—with moderate severity and 78.03% (*n* = 142)—with severe COVID-19, respectively. Inclusion/exclusion criteria have been described in our former publication [14].

Clinical and demographic characteristics of moderate–severe patients with COVID-19 is given in Table 1.

Moderate–severe patients in both groups did not differ in age, nor in SBP, DBP, and BMI (slightly worse indicators were observed in moderate severity). The sex distribution was parity: men/women (*n* = 97/*n* = 100), but the relative frequency of women dominated in severe COVID-19 and men in moderate COVID-19 by 17.45% (χ^2^ = 4.83; *p* = 0.028). The majority (87.31% of patients) received non-invasive oxygen therapy as a basic treatment: 100% (*n* = 142) of patients with severe COVID-19 and almost 55% (*n* = 30) of patients with moderate severity of the disease (χ^2^ = 73.93; *p* < 0.001). SpO2 in the group of patients with severe disease was lower than in those with moderate disease—by 10.0% (*p* = 0.025). The absolute number of smokers was equally divided between the groups (*n* = 25 in each), but the relative number prevailed more in moderate than in severe disease—by 27.85% (χ^2^ = 16.23; *p* < 0.001). The number of unvaccinated persons was almost twice as high as the number of vaccinated persons in each group, without a significant difference between them (χ^2^ = 0.4; *p* = 0.527).

The control group included 60 patients with mild COVID-19 who did not require hospitalization and were observed by a general practitioner. Their diagnoses was proved by a PCR-based method according to the protocol. Among patients with mild COVID-19, there were 40.0% (*n* = 24) men and 60.0% (*n* = 36) women, mean age 56.77 ± 5.67 yo; 66.67% (*n* = 40)—vaccinated and 33.33% (*n* = 20)—unvaccinated; 20 persons were smokers. Age and sex distributions did not differ from moderate–severe COVID-19 patients (*p* > 0.05).

GS-5734 (remdesivir), recommended by the FDA for the treatment of COVID-19 (2020) and approved by the WHO, was prescribed as the basic antiviral drug [13], according to the treatment regimen described in the drug’s instructions. Remdesivir was administered to hospitalized patients within the first 5 days of the onset of symptoms, or if the patient was hospitalized later, at any time if clinically indicated, as allowed by the clinical protocol. On the first day, a loading dose of 200 mg once daily (IV over 30–120 min), and from the second day, a maintenance dose of 100 mg once daily (IV over 30–120 min), were followed. The treatment duration was 5 days. Careful monitoring of remdesivir toxicity was performed: before starting therapy and daily during the use of remdesivir in adult patients, the estimated glomerular filtration rate (eGFR) was determined. Remdesivir was not used in patients with eGFR < 30 mL/min/1.73 m^2^. Such patients were not included in the study. Prior to treatment, the functional state of the liver was analyzed and monitored throughout the treatment period (if the increase in blood alanine aminotransferase (ALT) was more than 5 times, the drug was discontinued).

Blood samples were taken before treatment (on the first day of hospital admission) and before discharge from the hospital (on the 12–15th day of hospitalization).

The study was conducted in accordance with the moral and ethical standards of bioethics in accordance with the ICH/GCP, the Helsinki Declaration of Human Rights (1964), the Council of Europe Convention on Human Rights and Biomedicine (1997), and the current legislation of Ukraine. The study protocol was approved by the Bioethics Committee of Bukovinian State Medical University (Protocol No. 7 of April 2025). All subjects signed a written informed consent to participate in the study.

### 2.2. Laboratory and Clinical Data

A comprehensive laboratory examination was performed, including the determination of oxygen saturation (SpO2, %), transmembrane serine protease 2 (TMPRSS2), endothelin-1 (ET-1), interleukin-6 (IL-6), and procalcitonin (PCT).

SpO2 was measured using a pulse oximeter (Gamma Oxy Scan, SHENZHEN HOMED MEDICAL DEVICE, Shenzhen, China).

The serum levels of TMPRSS2, IL-6, ET-1 and PCT were determined by Enzyme Linked-Immuno-Sorbent Assay (ELISA) and Electrochemiluminescence Immunoassay (ECLIA), according to the Manufacturer’s Guidelines, with a highly sensitive TMPRSS2 ELISA KIT^®^ (MyBioSource, Inc. San Diego, CA, USA), Human Interleukin-6 ELISA KIT^®^ (Elabscience Biotechnology Inc., Houston, TX, USA), Human ET-1 ELISA KIT^®^ (Elabscience Biotechnology Inc., Houston, TX, USA), and PCT ECLIA KIT^®^ (Roche Elecsys^®^ PCT, Meylan, France) on “MAGLUMI 1000 Plus” device (“SNIBE”, Shenzhen, China).

ELISA principles for TMPRSS2, IL-6, and ET-1 evaluation: traditional microplate format using capture and detection antibodies. After sample incubation and washing, a substrate for the enzyme-conjugated secondary antibody yielded a colorimetric readout proportional to a reliable determined parameter (TMPRSS2, IL-6, ET-1). **Sensitivity**: ~0.1 ng/mL.

ECLIA principles for PCT determination: sandwich assay—one biotinylated antibody and one ruthenium-labeled antibody bind PCT. Streptavidin-coated microparticles capture the complex; electrical stimulation triggers light emission from the ruthenium label, quantified by a photomultiplier. Sensitivity: ~0.02 ng/mL.

D-dimer was determined by Electrochemiluminescence Immunoassay (ECLIA) with a highly sensitive Elecsys D-Dimer ECLIA KIT^®^ (Roche Diagnostics^®^, France) on “MAGLUMI 1000 Plus” device (“SNIBE”, China).

Fibrinogen was determined by the Clauss method with a quantitative FBG reagent Dia-FIB^®^ (Diagon GmbH, Budapest, Hungary) on the semi-automated coagulation analyzer DIAGON CoagM, manufactured by Diagon Ltd. (Budapest, Hungary). The method of Clauss measures the clotting time after adding a high concentration of thrombin to diluted plasma. The fibrinogen concentration in the plasma is inversely proportional to the clotting time. Sensitivity: ~0.05 g/L.

Biochemical parameters were evaluated on a biochemical automatic analyzer, “ACCENT 200” (CORMAY, Łomianki, Poland).

### 2.3. Identifying Genetic Polymorphisms

Genotyping of the FGB (rs1800790), NOS3 (rs2070744), and TMPRSS2 (rs12329760) genes was performed for 72 patients with moderate–severe COVID-19 (study group) and for 48 patients with a mild COVID-19 course (control group).

For the isolation of genomic DNA, peripheral blood leukocytes were acquired using a commercial kit (Thermo Scientific™ GeneJET™ Whole Blood Genomic DNA Purification Mini Kit, Thermo Fisher Scientific, Waltham, MA, USA). In detail, 200 μL of whole blood from each participant was digested with proteinase K followed by the addition of lysis buffer. The next steps included washing and elution of the purified DNA.

Real-time polymerase chain reaction (RT-PCR) was used to genotype the polymorphism of the FGB (rs1800790), NOS3 (rs2070744), and TMPRSS2 (rs12329760) genes. For this purpose, the CFX96™ real-time PCR detection system (Bio-Rad Laboratories, Inc., USA) was used. Specific TaqMan™ kits were used for each target SNP. Genotyping was performed using TaqMan^®^ probes and TaqMan^®^ Genotyping Master Mix (4371355) in combination with the CFX96™ Real-Time PCR Detection System, as described in our former publication [14]. The PCR protocol strictly followed the manufacturer’s instructions (Applied Biosystems, Foster City, CA, USA). The TaqMan^®^ Genotyping Master Mix includes AmpliTaq Gold^®^ DNA polymerase, dNTPs, ROX™ reference dye, and optimized reaction buffers. For the identification of gene alleles, TaqMan^®^ probes were used, which are allele-specific oligonucleotides with reporter dyes (VIC^®^ for allele 1 and 6-FAM™ for allele 2) attached to the 5′ end and a non-fluorescent quencher (NFQ) at the 3′ end. Genomic DNA (10 μL) was amplified in a reaction mixture containing primers, probes, master mix, and target DNA. For genotyping, allele discrimination based on relative fluorescence units (RFUs) was used, employing CFX-Manager™ software (version 3.1). The PCR cycle conditions were as follows: initial denaturation: 95 °C for 10 min; amplification cycles (49 cycles); denaturation: 95 °C for 15 s; annealing: 60 °C for 1:10 min; final melting curve analysis: increase in temperature to 95 °C. Genotype determination was based on melting curve analysis using CFX96™ Real-Time PCR Basic software version 3.1 (Bio-Rad Laboratories, Inc., Hercules, CA, USA).

### 2.4. Statistical Analysis

Statistically, the results were processed in accordance with modern requirements, using the Statistica 13.0 program (StatSoft Inc., Tulsa, OK, USA, license number JPZ804I382130ARCN10-J). Pearson’s criterion (χ^2^) was used for the genotype distribution comparison. The reliability of data for independent samples with an array distribution close to normal was calculated by the Student’s *t*-test, and in case of uneven distribution, by the Wilcoxon–Mann–Whitney U test. Differences were considered significant at *p* < 0.05.

## 3. Results

In this study, we found that smokers and men are at a lower risk of having a more severe course of COVID-19 (OR = 0.26; OR 95%CI: 0.13–0.51; *p* < 0.001 and OR = 0.49; OR 95%CI: 0.26–0.93; *p* = 0.038), whereas the risk of a moderate severity of COVID-19 increases by twofold in men (OR = 2.02; OR 95%CI: 1.06–3.80; *p* = 0.041) and almost fourfold in smokers (OR = 3.90; OR 95%CI: 1.97–7.73; *p* < 0.001) (Table 1). On the contrary, the risk of having a severe course of COVID-19 is doubled in women (OR = 2.03; OR 95%CI: 1.07–3.84; *p* = 0.021).

The distribution of alleles and genotypes of the FGB (rs1800790), NOS3 (rs2070744), and TMPRSS2 (rs12329760) genes in patients with COVID-19 is shown in Table 2.

The distribution of FGB gene (rs1800790) genotypes between the groups differed (Pearson χ^2^ = 7.87; *p* = 0.005, without adjustment for continuity): the relative frequency of the wild-type G-allele significantly prevailed in the study group, and the A-allele, on the contrary, in the control group by 13.19% (χ^2^ = 4.36; *p* = 0.037). The relative frequency of the GG genotype carriers dominated in the study group by 25.0% (χ^2^ = 7.50; *p* = 0.006), but the AG genotype, on the contrary, dominated in the control group by 23.61% (χ^2^ = 6.43; *p* = 0.011). The G-allele prevailed in both groups over the A-allele, but reliably only in patients with moderate–severe COVID-19—by 38.88% (χ^2^ = 43.56; *p* < 0.001).

Among the alleles of the endothelial nitric oxide synthase (NOS3) gene dpSNP, rs2070744, in patients with COVID-19 in both the study and control groups, the wild T-allele dominated over the C-allele as follows: in the study group by 19.44% (χ^2^ = 10.89; *p* = 0.001), and in the control group by 18.75% (χ^2^ = 6.75; *p* = 0.009). The distributions of genotypes and alleles between the groups did not differ.

The frequency of the C-allele of the TMPRSS2 gene (rs12329760) in the homozygous state was higher among patients with moderate–severe COVID-19 than in the control group with a mild COVID-19 course by almost 18.06% (χ^2^ = 3.76; *p* = 0.05). In contrast, the frequency of the T-allele (especially the TC genotype) dominated, but not significantly, in the control group by 10.49% (χ^2^ = 2.76; *p* = 0.065) and 15.97% (χ^2^ = 3.01; *p* = 0.061). In both groups, the wild-type C allele prevailed over the mutational T allele by 48.06% (χ^2^ = 64.22; *p* = 0.001) and 27.08% (χ^2^ = 14.08; *p* = 0.001), respectively. In general, there were no statistically significant differences in the distribution of genotypes between the groups (χ^2^ = 2.62; *p* = 0.105).

The analysis of the inheritance patterns of susceptibility to COVID-19, taking into account the 455G > A polymorphism of the FGB gene (dpSNP: rs1800790), is shown in Table 3.

The probability of the three models’ in susceptibility to the development of moderate–severe COVID-19, taking into account the 455G > A polymorphism of the FGB gene, was determined as follows: codominant (*p* = 0.02), dominant (*p* = 0.01), superdominant (*p* = 0.01), and additive (*p* = 0.03), of which the dominant model with the lowest Akaike coefficient (AC = 16.03) is the most effective. According to these models, the lowest pathology development is expected in carriers of the mutational A-allele of the FGB gene (OR = 0.31–0.54; 95% CI: 0.13–0.95; *p* ≤ 0.03–0.01).

The association of the T-786C polymorphism of the NOS3 gene (dpSNP: rs2070744) with the onset of COVID-19 was analyzed using binary logistic regression (Table 4).

No model was statistically significant for the allelic state of the NOS3 gene rs2070744. The lowest error of out-of-sample prediction of the moderate–severe disease course in the population (Akaike’s coefficient) is inherent in the superdominant and subdominant models (AC = 16.17 and 16.19; *p* = 0.05).

The analysis of inheritance models of susceptibility to moderate–severe COVID-19, taking into account the Val160Met C/T polymorphism of the TMPRSS2 gene (rs12329760), is shown in Table 5.

The analysis of genetic models of inheritance showed a tendency to the moderate–severe COVID-19 in the dominant model, where the presence of a T-allele increases the likelihood of illness more than 2 times (OR = 2.08; OR95%CI: 1.0–4.45; *p* = 0.049). This model was statistically significantly the most effective, with the lowest coefficient of Akayke (CA = 15.69). A similar tendency was confirmed in the super-dominant model, where the CT-genotype availability increased the risk of moderate–severe COVID-19 in the surveyed population to almost double but was poor (OR = 1.92; OR95%CI: 0.92–4.08; *p* = 0.08).

The relative frequency of the SNP genes FGB (RS1800790), eNOS (RS2070744), TMPRSS2 (RS12329760) is not dependent on the severity of the COVID-19 clinical course and does not affect its risk (Table 6).

Epidemiologic analysis confirmed that the risk of severe COVID-19 doubles in women (OR: 2.03; OR 95%CI: 1.07–3.84; *p* = 0.021) and is associated with oxygen therapy (OR: 22.83; OR 95%CI: 8.08–64.49; *p* < 0.001). At the same time, smokers and men were found to have a significantly lower rate of severe COVID-19 (OR = 0.26; OR 95%CI: 0.13–0.51; *p* < 0.001 and OR = 0.49; OR 95%CI: 0.26–0.93; *p* = 0.038).

Instead, the risk of moderate COVID-19 severity is double in men (OR: 2.02; OR 95%CI: 1.06–3.80; *p* = 0.041) and increased almost 4 times in smokers (OR = 3.90; OR 95%CI: 1.97–7.73; *p* < 0.001).

To evaluate the effect of treatment, we analyzed the inflammatory response (IL-6), fibrinogen, D-dimers, ET-1 blood level, SpO2, and blood TMPRSS2 (Table 7). Under the influence of complex antiviral treatment, a significant decrease in TMPRSS2 and IL-6 was found in patients with moderate COVID-19, with decreases of 16.38% (*p* = 0.014) and 40.62% (*p* < 0.001); in patients with severe COVID-19, the decreases were 11.30% (*p* = 0.049) and 48.24% (*p* < 0.001), respectively. Moreover, the fibrinogen concentration likewise diminished after treatment in both groups, but this was not significant. Meanwhile, D-dimers statistically reduced under the influence of treatment in both groups: in moderate severity by 39.56% (*p* * = 0.015), and in severe COVID-19 by 41.86% (*p* * = 0.003), but D-dimers continued to exceed control values (*p* < 0.05).

There were no significant changes in the concentration of endothelin-1 in the blood, which did not depend on the severity of the disease but reached several times higher than the control group at 2.51–3.32 times (*p* < 0.001), which may have negative consequences in terms of the appearance of long-standing COVID-19 in the future from the cardiovascular system, or any organ and/or system where the vascular endothelium suffers most (formation of endothelial dysfunction). PCT, as a marker of bacterial burden, mainly decreased under the influence of treatment, as all patients with moderate and severe conditions received additional antibacterial therapy according to the indications according to the treatment protocol: in moderate severity it decreased by 1.93 times (*p* < 0.001), and in severe COVID-19 by 2.33 times (*p* < 0.001), respectively. The SpO2 level significantly improved in all observation groups (by 6.66%; *p* < 0.05 and 14.81%; *p* < 0.001), which made it possible to discharge patients home in a compensated state for outpatient rehabilitation.

## 4. Discussion

Previous studies have found significant differences in allele frequencies in the ACE2 and TMPRSS2 genes (receptor and coreceptor genes for SARS-CoV-2, respectively) between patients with COVID-19 and the general population [15,16]. However, these studies focused only on the regions of these genes. Regulatory regions have not been properly studied. Polymorphic gene variants in regulatory regions, especially enhancers, can disrupt regulatory function, affect the expression of other genes, and thus determine susceptibility to viral infection and the severity of the infectious disease [17,18].

In our study, we found that the distribution of FGB (rs1800790) gene genotypes between groups of patients with coronavirus infection and healthy individuals differed, with the relative frequency of the wild-type G-allele and GG genotype being significantly higher in the patient group; the A-allele, on the contrary, being higher in the control group; and the G-allele prevailing in both groups over the A-allele. It was determined that the mutation of the NOS3 gene (T-786C, rs2070744) in the homozygous state occurred in almost every 5th subject, with dominance of the wild-type T allele over the mutant allele in both groups. In turn, the mutational T-allele of the TMPRSS2 gene (rs12329760) showed no significant differences in frequency between patients with COVID-19 and healthy controls, with a higher frequency of the CC genotype among patients than in the control group.

Given the wide variability in individual responses to SARS-CoV-2 infection, it is important to understand whether genetic and biological predictors can predispose to infection or determine its severity, including the development of systemic coagulopathy and thrombosis. Cases have been described whereSARS-CoV-2, on the contrary, can become a suppressor of anticoagulant or fibrinolytic gene expression [19]. Separate studies in Bergamo (Italy) have investigated the involvement of some gene SNPs in this condition (FII rs1799963, FV rs6025, FV rs118203907, FXIIIA1 rs5985, FGB rs1800790, MTHFR rs1801131, MTHFR rs1801133) [20]. A strong association of the FGB rs1800790 (G > A) gene with higher fibrinogen levels in A-allele carriers [21]—even under low pro-inflammatory triggers in individuals with normal levels of C-reactive protein (CRP) and IL6 [22]—and an effect on the transcription rate of the β-chain of fibrinogen [23] have been described. Schwedler C et al. [24] found that combinations of wild-type alleles of the fibrinogen gene (G-allele), factor XIII A-subunit (F13A), and α2-antiplasmin (A2AP), which promote the formation of dense fibrin gels with high antifibrinolytic capacity (e.g., carrying the A-allele of the FGB rs1800790 gene in the F13A 34Val/Val, or A2AP 6Arg/Arg wild-type variants), are associated with reduced inflammation. Fibrinogen is an acute-phase protein; its increase has been observed in bacterial and viral infection, and this increase can contribute to trapping pathogens and limiting their diffusion. In some research, the GG allele of FGB is associated with lower levels of fibrinogen than the A-allele (2.87  ±  0.18 mg/dl vs. 3.29  ±  0.38 mg/dl, *p*  <  0.001) [25]. While in another study, the carriage of the FGBrs1800790 A allele exhibited an unreliable effect on fibrinogen concentration, or even an opposite effect—wildtype G-allele carriers (F13A34Val/Val i A2AP 6Arg/Arg) of the FGB rs1800790 gene had higher fibrinogen concentrations than the minor A-allele (4.62 ± 1.23 vs. 4.45 ± 1.12; *p* < 0.01 and 4.56 ± 1.25 vs. 4.44 ± 1.07; *p* < 0.04, respectively). In our study, the risk of severe COVID-19 is lower in minor A-allele carriers of the FGB rs1800790 gene, which, in our opinion, is due to a higher level of fibrinogen in these patients, which inhibits/limits the spread of SARS-CoV-2 infection and is generally consistent with the literature data [26,27]. In this perspective, we will provide data on the dependence of fibrinogen and coagulation parameter changes related to genes’ polymorphisms.

Using binary logistic regression, we confirmed a low probability of COVID-19 developing in carriers of the mutational A-allele of the FGB gene within the codominant, dominant, superdominant, and additive models of pathology inheritance, and we also recorded an increased risk of COVID-19 in carriers of the G-allele (especially the GG genotype) of the FGB gene (rs1800790), with a protective role of the A-allele and AG genotype, respectively. In turn, the alleles and genotypes of the NOS3 gene (T-786C, rs2070744) were not predictors of COVID-19 in the study population. In contrast, the presence of a mutational T-allele of the TMPRSS2 gene (rs12329760) in the genotype increases the likelihood of the disease by more than 2 times, which confirms the role of the TMPRSS2 gene in increasing the likelihood of the clinical development of COVID-19.

In some studies, it was proved that COVID-19 is generally less severe in smokers compared to non-smokers [28], although discrepancies and conflicting reports are likewise present [29]. In our study, we found that smokers and men were at a lower risk of having a more severe course of COVID-19, whereas the risk of moderate-severity COVID-19 increased twofold in (*p* = 0.041), and almost fourfold in smokers (*p* < 0.001). On the contrary, the risk of having a severe course of COVID-19 was doubled in women (*p* = 0.021). This may be explained by the fact that all the smokers in our research were men, and no women with severe COVID-19 in this study were smokers. Therefore, we hypothesize about the protective role of nicotine in this case. But the postulated defensive effects of nicotine, which may potentially reduce the risk of a “cytokine storm” in infected individuals with less severe disease, deserve further attention and analysis in controlled clinical trials.

The development of antiviral drugs against SASR-CoV-2 is focused on preventing hospitalization, intubation, or death in patients with COVID-19 at high risk of disease progression [30]. Remdesivir, the only FDA-approved drug for the treatment of patients with COVID-19, showed significant benefits in clinical improvement and viral suppression without reducing mortality among unvaccinated patients during the initial wave of the generic SARS-CoV-2 COVID-19 strain [31,32]. RNA-dependent RNA polymerase can utilize remdesivir triphosphate as a substrate, leading to the incorporation of remdesivir monophosphate (RMP) into the growing RNA product. Once RMP is incorporated, RNA-dependent RNA polymerase (RdRp) elongates the RNA for an additional three nucleotides before it stops. The mechanism is specific to coronaviruses [33]. Whereas these studies explain how RMP is incorporated into RNA instead of AMP, they do not explicate how remdesivir inhibits RdRp, since RdRp stops only after adding three additional nucleotides to the RNA.

According to the results of the study, we found that the administration of the antiviral drug GS-5734 (remdesivir) and anti-inflammatory therapy reduces the level of TMPRSS2 in the blood, with the most significant changes observed in patients with moderate-severity COVID-19. This proves the direct influence of SARS-CoV-2 viral load on TMPRSS2 synthesis, which can be explained by its incorporation into the GS-5734 system, reducing the stimulation of the adhesion protein on the airway epithelial cells, which reduces the need for its production and expression of the corresponding gene. According to another hypothesis, which we believe is less likely to have an impact, general anti-inflammatory pathogenetic therapy (some patients received methylprednisolone and dexamethasone, antiviral detoxification, membrane-stabilizing therapy according to the protocol) reduces the stress of the monocyte–macrophage immune response and the oxidative stress redox system. Furthermore, several commonly used therapies for COVID-19 patients with type 2 diabetes, such as glucose-lowering and anti-inflammatory agents, may indirectly modulate the gut microbiota composition and systemic immune responses, potentially influencing disease progression and recovery trajectory [34,35,36,37].

Regarding other laboratory results, we found that endothelin-1 did not change under the influence of the proposed treatment. Accordingly, it requires further research, as some patients had concomitant cardiovascular disease. High levels of ET-1 may be associated with its high expression in the blood and vascular endothelium and could affect the long-term effects of longitudinal vascular injury [38,39,40]. It was also found that the level of procalcitonin decreased significantly because all patients received antibacterial therapy in parallel, in accordance with the results of previous studies [41,42,43]. It is important to note that IL-6, a pro-inflammatory marker, in turn, responded best to treatment but remained elevated several more times compared to controls [44,45,46,47]. A course of hospital treatment was accompanied by a decrease in the fibrinogen level (*p* > 0.05) and a significant reduction in D-dimers, but they continued to exceed the reference values. This will require a lasting administration of antiplatelet therapy, or oral anticoagulants, in some patients, according to the protocol and depending on the coagulation condition. Therefore, continuation of nonspecific anti-inflammatory therapy during the outpatient phase is recommended to mitigate the long-term effects of chronic COVID-19, as sustained low-grade inflammation may contribute to prolonged symptoms, delayed tissue recovery, and the development of post-acute sequelae such as fatigue, dyspnea, or cardiovascular complications [48,49,50]. These findings emphasize the complexity of inflammatory regulation in COVID-19 and highlight the need for tailored therapeutic approaches [51,52,53,54].

Recent studies highlight the role of gene expression modulation in COVID-19 outcomes, particularly in patients with metabolic comorbidities [55,56]. However, the variability in individual immune responses suggests that a one-size-fits-all approach may be insufficient for optimal recovery [57,58,59]. Personalized treatment strategies considering patient-specific factors are therefore critical [60,61,62]. Genetic variants in IFNAR2, OAS1, OAS3, and ACE2 may influence the effectiveness of Paxlovid, especially in the presence of MAFLD, supporting a personalized treatment approach [63,64,65]. Moreover, integrating multi-omics data—including genomics, transcriptomics, and proteomics—could improve patient stratification and guide precision medicine initiatives [66,67,68,69]. Emerging biomarkers may also help to predict disease trajectory and inform therapeutic decisions in real time [53,70,71,72,73,74]. Understanding the interplay between host genetics and viral pathogenesis remains a key to identifying high-risk populations [75,76,77,78]. Such approaches could also facilitate early interventions and reduce the burden of post-acute complications [58,79,80]. Targeted therapeutic interventions may offer improved outcomes in genetically predisposed individuals [77,81,82,83]. Stratifying patients based on their genotypic profiles could enhance the precision of antiviral and supportive therapies, minimizing adverse effects and maximizing efficacy [84,85,86]. Further longitudinal studies are essential to better understand persistent immunological alterations and optimize patient management strategies.

## 5. Conclusions

The highest probability of developing moderate and severe clinical courses of COVID-19 among residents of Central Ukraine was found in G-allele carriers (especially the GG genotype) of the FGB gene (rs1800790) and carriers of the T-allele TMPRSS2 gene (rs12329760).

Administration of the antiviral drug GS-5734 (remdesivir) and anti-inflammatory therapy reduce the blood level of TMPRSS2 in moderate-course and of IL-6 in severe-course COVID-19. The proposed treatment does not significantly affect the concentration of endothelin-1, but a decrease in procalcitonin associated with additional antibacterial drugs was observed, especially in severe COVID-19, in addition to reduced D-dimers and procalcitonin values in all patients. The proposed treatment does not significantly affect the endothelin-1 concentration or fibrinogen level.

## Figures and Tables

**Table 1:** Clinical and demographic characteristics of moderate–severe patients with COVID-19.

Individual Factors	Moderate Course *n* = 55	Severe Course *n* = 142	χ^2^	*p*
Age, years (M ± m)	63.97 ± 10.58	68.78 ± 11.09	-	0.140
Women, *n* = 100 (%)	21 (38.18)	79 (55.63)	4.83	0.028
Men, *n* = 97 (%)	34 (61.82)	63 (44.37)		
Vaccinated, *n* = 75	19 (34.55)	56 (39.44)	0.4	0.527
Unvaccinated, *n* = 122	36 (65.45)	86 (60.56)		
Non-invasive oxygen therapy, *n* = 172	30 (54.56)	142 (100.0)	73.93	<0.001
No oxygen therapy, *n* = 25	25 (45.45)	0		
SBP, mm/Hg	148.47 ± 3.70	142.78 ± 3.66	-	0.077
DBP, mm/Hg	88.69 ± 3.19	87.92 ± 3.17	-	0.184
BMI, kg/m^2^	30.23 ± 1.15	29.09 ± 0.88	-	0.211
SpO2, %	0.90 ± 0.04	0.81 ± 0.05	-	0.025
T2DM, *n* = 52	13 (23.64)	39 (27.46)	0.3	0.584
Smoking, *n* = 50	25 (45.45)	25 (17.60)	16.23	<0.001

Notes. T2DM—type 2 diabetes mellitus; SBP, DBP—systolic, diastolic blood pressure; BMI—body mass index.

**Table 2:** Distribution of genes FGB (rs1800790), NOS3 (rs2070744), and TMPRSS2 (rs12329760) in observed population.

Polymorphic Variants of Genes			Study Group, *n* = 72 (%)	Control Group, *n* = 48 (%)	χ^2^	*p*
***FGB gene (455G > A; rs1800790)***						
*FGB* (455*G > A*), *n* (%)	*GG*		36 (50.0)	12 (25.0)	7.50	0.006
	*AG*		28 (38.89)	30 (62.50)	6.43	0.011
	*AA*		8 (11.11)	6 (12.50)	0.05	0.823
χ^2^; *p*			χ^2^ = 5.84 *; *p* = 0.016		-	
*FGB* (455*G > A*), *n* (%)	Allele *G*		100 (69.44)	54 (56.25)	4.36	0.037
	Allele *A*		44 (30.56)	42 (43.75)		
***NOS3 gene (T-786C; rs2070744)***						
*NOS3* (*T-786C*), *n* (%)		*TT*	28 (38.89)	18 (37.50)	0.02	0.887
		*TC*	30 (41.67)	21 (43.75)	0.05	0.823
		*CC*	14 (19.44)	9 (18.75)	0.01	0.920
χ^2^; *p*			χ^2^ = 0.05; *p* = 0.823		-	-
*NOS3* (*T-786C*), *n* (%)		Allele *T*	86 (59.72)	57 (59.37)	0	1.0
		Allele *C*	58 (40.28)	39 (40.62)		
***TMPRSS2 gene (Val160Met C/T; rs12329760)***						
*TMPRSS2* (*Val160Met C/T*), *n* (%)		*CC*	40 (55.56)	18 (37.50)	3.76	0.05
		*CT*	26 (36.11)	25 (52.08)	3.01	0.061
		*TT*	6 (8.33)	5 (10.42)	0.04	0.467
χ^2^; *p*			χ^2^ = 2,62; *p* = 0.105		-	-
*TMPRSS2* (*Val160Met C/T*), *n* (%)		Allele *C*	106 (74.03)	61 (63.54)	2.76	0.065
		Allele *T*	38 (25.97)	35 (36.46)		

Notes. *—for df = 1 χ^2^ Yates with continuity correction (χ^2^ Pearson without continuity correction = 7.87; *p* = 0.005); χ^2^—Pearson coefficient.

**Table 3:** Models of inheritance of susceptibility to moderate–severe COVID-19 based on the 455G > A polymorphism of the FGB gene (dpSNP: rs1800790).

Genotypes	Study Group, *n* = 72 (%)	Control Group, *n* = 48 (%)	OR [95% CI]	*p*	AC
The codominant model					
GG	36 (50.0)	12 (25.0)	1.00	0.02	17.68
AG	28 (38.89)	30 (62.50)	0.31 [0.13–0.70]		
AA	8 (11.11)	6 (12.50)	0.44 [0.13–1.59]		
The dominant model					
GG	36 (50.0)	12 (25.0)	1.00	0.01	16.03
AG + AA	36 (50.0)	36 (75.0)	0.33 [0.15–0.73]		
Recessive model					
GG + AG	64 (88.89)	42 (87.50)	1.00	0.82	23.70
AA	8 (11.11)	6 (12.50)	0.87 [0.28–2.83]		
Super-dominant model, df = 2					
GG + AA	44 (61.11)	18 (37.50)	1.00	0.01	17.27
AG	28 (38.89)	30 (62.50)	0.38 [0.18–0.80]		
Additive model					
GG	36 (50.0)	12 (25.0)	1.00	0.03	19.14
2AA + AG	44	42	0.54 [0.30–0.95]		

Notes. OR—odds ratio; CI—confidence interval; df—degrees of freedom; (in the superdominant model df = 2, in other models df = 1); AC—Akaike’s coefficient.

**Table 4:** Models of inheritance of susceptibility to moderate–severe COVID-19 based on the T-786C polymorphism of the NOS3 gene (dpSNP: rs2070744).

Genotypes	Study Group, *n* = 72 (%)	Control Group, *n* = 48 (%)	OR [95% CI]	*p*	AC
		*The codominant model*			
*TT*	28 (38.89)	18 (37.50)	1.00	0.97	18.17
*TC*	30 (41.67)	21 (43.75)	0.92 [0.40–2.07]		
*CC*	14 (19.44)	9 (18.75)	1.0 [0.36–2.85]		
		*The dominant model*			
*TT*	28 (38.89)	18 (37.50)	1.00	0.88	16.19
*TC + CC*	44 (61.11)	30 (62.50)	0.94 [0.44–2.0]		
		*Recessive model*			
*TT + TC*	58 (80.56)	39 (81.25)	1.00	0.92	16.21
*CC*	14 (19.44)	9 (18.75)	1.05 [0.45–2.73]		
		*Super-dominant model, df = 2*			
*TT + CC*	42 (58.33)	27 (56.25)	1.00	0.82	16.17
*TC*	30 (41.67)	21 (43.75)	0.92 [0.44–1.93]		
		*Additive model*			
*TT*	28 (38.89)	18 (37.50)	1.00	0.96	16.22
2*CC + TC*	58	39	0.99 [0.60–1.93]		

Notes. OR—odds ratio; CI—confidence interval; df—degrees of freedom; (in the superdominant model df = 2, in other models df = 1); AC—Akaike’s coefficient.

**Table 5:** Models of inheritance of susceptibility/susceptibility to moderate–severe COVID-19 taking into account Val160Met C/T polymorphism of the TMPRSS2 gene (rs12329760).

Genotypes	Study Group, *n* = 72 (%)	Control Group, *n* = 48 (%)	OR [95% CI]	*p*	CA
*The codominant model*, df = 1					
*CC*	40 (55.56)	18 (37.50)	1.00	0.15	17.65
*CT*	26 (36.11)	25 (52.08)	2.14 [0.98–4.73]		
*TT*	6 (8.33)	5 (10.42)	1.85 [0.48–6.95]		
*The dominant model*, df = 1					
*CC*	40 (55.56)	18 (37.50)	1.00	0.049	15.69
*CT+ TT*	32 (44.44)	30 (62.50)	2.08 [1.0–4.45]		
*Recessive model,* df = 1					
*CC + CT*	66 (91.67)	43 (89.58)	1.00	0.70	19.33
*TT*	6 (8.33)	5 (10.42)	1.28 [0.35–4.50]		
*Super-dominant model*, df = 2					
*CC + TT*	46 (63.89)	23 (47.92)	1.00	0.08	16.48
*CT*	26 (36.11)	25 (52.08)	1.92 [0.92–4.08]		
*Additive model*, df = 1					
*CC*	40 (55.56)	18 (37.50)	1.00	0.10	16.72
2*TT + CT*	38 (52.78)	35 (72.92)	1.61 [0.92–2.88]		

Notes. OR—odds ratio; CI—confidence interval; df—degrees of freedom; CA—Akaike’s coefficient.

**Table 6:** Distribution of genotypes of polymorphism of FGB genes (455G > A; rs1800790), ENOS (786T > C; rs2070744), and TMPRSS2 (Val160Met C/T; rs12329760) in moderate–severe COVID-19 patients.

Genes		Genotypes	Moderate Course, *n* = 36 (%)	Severe Course, *n* = 36 (%)	χ^2^		*p*
In general, *n* = 197 (%)			55 (27.92)	142 (72.08)	78.64		<0.001
	***FGB (rs1800790) gene***						
*FGB* (455G > A), *n* = 72 (%)		*GG*	18 (50.0)	18 (50.0)	0		1.0
		*GA + AA*	18 (50.0)	18 (50.0)			
	***eNOS (rs2070744) gene***						
eNOS (786T > C), *n* = 72 (%)		*TT*	16 (44.44)	12 (33.33)	1.1		0.294
		*CT*	13 (36.11)	17 (47.22)			
		*CC*	7 (19.44)	7 (19.44)			
	***TMPRSS2 (rs12329760) gene***						
TMPRSS2 (C/T), *n* = 72 (%)		*CC*	20 (55.56)	20 (55.56)	0		1.0
		*CT + TT*	16 (44.44)	16 (44.44)			

**Table 7:** Laboratory findings of inflammatory activity, SpO2, Fibrinogen, D-dimer and TMPRSS2 contents in patients with COVID-19 before/after treatment.

Laboratory Findings		Control	Moderate Course	Severe Course
TMPRSS2, ng/mL	Before treatment	1.81 ± 0.12	2.87 ± 0.18 *p* < 0.001	2.30 ± 0.19 *p* = 0.003; *p* # < 0.001
	After treatment		2.40 ± 0.11 *p* = 0.003; *p* * = 0.014	2.04 ± 0.06 *p* = 0.043; *p*# = 0.002; *p* * = 0.049
ET-1, pg/mL	Before treatment	4.03 ± 0.55	13.37 ± 2.97 *p* < 0.001	10.81 ± 3.53 *p* = 0.047
	After treatment		11.56 ± 1.62 *p* < 0.001	10.11 ± 0.95 *p* = 0.002
IL-6, pg/mL	Before treatment	7.79 ± 1.26	42.86 ± 7.48 *p* < 0.001	100.79 ± 4.96 *p*, *p* # < 0.001
	After treatment		25.45 ± 3.26 *p*, *p* * < 0.001	52.17 ± 2.85 *p*, *p* #, *p* * < 0.001
PCT, ng/mL	Before treatment	0.1 ± 0.0001	0.29 ± 0.06 *p* < 0.001	0.28 ± 0.06 *p* < 0.001
	After treatment		0.15 ± 0.02 *p*, *p* * < 0.001	0.12 ± 0.02 *p*, *p* * < 0.001
SpO2, %	Before treatment	0.98 ± 0.01	0.90 ± 0.04 *p* < 0.001	0.81 ± 0.05 *p* < 0.001; *p* # = 0.025
	After treatment		0.96 ± 0.01 *p* * = 0.048	0.93 ± 0.02 *p* * < 0.001 *p* = 0.013; *p* # = 0.051
Fibrinogen, g/l	Before treatment	3.52 ± 0.22	4.84 ± 0.50 *p* < 0.001	5.20 ± 0.37 *p* < 0.001
	After treatment		4.30 ± 0.35 *p* = 0.031	4.71 ± 0.23 *p* = 0.003
D-dimer, mg/l FEU	Before treatment	0.34 ± 0.05	0.91 ± 0.13 *p* < 0.001	0.86 ± 0.10 *p* < 0.001
	After treatment		0.55 ± 0.10 *p* = 0.032; *p* * = 0.015	0.50 ± 0.08 *p* = 0.045; *p* * = 0.003

Notes. TMPRSS2: transmembrane serine protease 2; ET-1: endothelin-1; IL-6: interleukin-6; PCT: procalcitonin; *p*: significance of differences with the control group; *p* #: significance of differences with moderate severity of COVID-19; *p* *: significance of differences with the pretreatment state in each group separately.

## Data Availability

Data are contained within the article.
